# The Use of the Alberta Infant Motor Scale (AIMS) as a Diagnostic Scale for Infants with Autism

**DOI:** 10.3390/diagnostics13061045

**Published:** 2023-03-09

**Authors:** Mooly Kochav-Lev, Odeya Bennett-Back, Meir Lotan, Chen Stein-Zamir

**Affiliations:** 1 Child Development Institute, Meuhedet Health Services, Jerusalem 95464, Israel; 2 Department of Physiotherapy, Ariel University, Ariel 40700, Israel; 3 Braun School of Public Health and Community Medicine, The Hebrew University, Jerusalem 91120, Israel; 4 Jerusalem District Health Office, Israel Ministry of Health, Jerusalem 9101002, Israel

**Keywords:** autism, developmental physical therapy, AIMS

## Abstract

Autism spectrum disorder (ASD) is a group of developmental disabilities presenting difficulties in social interaction and language and an increased occurrence of cognitive, sensory, and motor gaps. Early intervention has been reported to improve the function of children with ASD. However, motor screening for children with ASD is difficult, as there are no specific tools for identifying this specific population. This study reports the results of using the Alberta Infant Motor Scale (AIMS), which assesses gross infant motor skills from ages 0 to 18 months, as a screening tool for detecting motor developmental delay (MDD) in small children with ASD. Methods: This retrospective cohort study included all children registered at one health care organization in Israel born between 2011 and 2017 (N = 240,299). Early childhood MDD was defined as having at least one recorded developmental physiotherapy (DPT) visit before the age of 2 years. Reasons for referral to DPT and the results of using AIMS as an appropriate tool for revealing developmental delays in infants with ASD are presented. Results: ASD diagnosis was reported in 1821 children (prevalence rate 0.75%). Of those, 388 (odds ratio 4.1, 95% CI 3.6–4.6) children were referred to DPT. Children with ASD mostly received DPT for motor delays (46.19%), torticollis (19.52%), developmental delay (15.48%), and preterm birth (7.38%). The use of AIMS as an early detection tool suggests that more than 87% of children with ASD and MDD present with a developmental delay or risk for one when using this scale. Conclusions: The prevalence of ASD among children referred to DPT for MDD is higher than its prevalence within the general population. The most common reasons for a child with ASD to be referred for DPT services are MMDs. AIMS was found to be a sensitive tool to pinpoint relevant candidates for ASD screening among children treated in DPT. Possible effects of the study: The use of AIMS as a relevant assessment scale for this group of clients is recommended. Training DPTs in identifying initial ASD signs and developing their clinical reasoning abilities will increase the chance of implementing early intervention with this group of clients.

## 1. Introduction

Motor development [1] is the process by which infants and later children learn to move their body parts and control their muscles so that they can perform a variety of motor skills, such as lifting the head, crawling, sitting, standing, and walking. This developmental process is accompanied by physical and cognitive development in which a baby learns how to move his or her body. The acquisition of motor skills is a basis for mobility and consequently a basis for exploring the world of the infant and then the child in a way that affects his or her sensory, cognitive, and emotional development and, finally, involvement in family life and in the physical and social environment. When a child’s motor development rate does not follow the typical pattern, it leads to a delay in motor development, which is known as motor developmental delay (MDD). The prevalence of MDD was shown by studies that tested the reliability of the Alberta Infant Motor Scale—AIMS which assesses motor development [2] (will be detailed later). A study conducted in the Netherlands in 2019 among 499 participants found a rate of 16% of infants with MDD (score below the 5th percentile) [3]. Another study conducted in Brazil in 2013 among 795 participants found a rate of 10.4% of infants with MDD (score below the 5th percentile) and 24.2% with suspicion of MDD (score below the 25th percentile) [4].

An additional study examined the prevalence of developmental delay in the US in 2008 among approximately 4 million children. The study used the Bayley Short Form-Research (BSF-R) test, which includes a mental component and a motor component. MDD was defined as a rating of at least one standard deviation below average in the motor component. In the study, it was found that at the age of 9 months, MDD was found to be 16% and at 24 months, MDD was found to be 14.7% [5].

Babies with MDD are diverse in their degree of severity and developmental trajectory [6]. Some children with MDD will “catch up” and reach the remaining milestones on time. In contrast, other children will later be diagnosed with a motor disability such as cerebral palsy or DCD. MDD is the initial and clearest sign in the case of general developmental delay. In babies, motor activity is a “window” to early development. Furthermore, through early detection of some of the babies, it is possible to influence their health, development, functioning, and family planning. 

Autism spectrum disorder (ASD) [7] is a neurological-developmental disorder that manifests in almost all areas of a child’s development. It is now commonly referred to as a wide range of communication disorders that include difficulties in social interaction and language. It finds expression in an extensive repertoire of the child’s emotional abilities as well as in behaviors with a patterned tone and lack of flexibility that are expressed in play and language. ASD children also present a high rate of challenges in regard to cognitive, motor, and sensory abilities [8].

Over the past 50 years, ASD has gone from a rare problem with a specific diagnosis appearing in childhood to a common and diverse condition, recognized by research and present throughout the years of life.

In 2010, 52 million cases of ASD were reported worldwide, a prevalence of 7.6 per 1000 or one in 132 people [9]. The Center for Disease Control and Prevention (CDC) reported a prevalence of ASD of 1 in 44 or 2.3% [10]. A study conducted in Israel in 2015 based on the National Insurance data found an ASD prevalence rate of 0.64% [11].

In the last 10 years, dozens of studies that examined the presence of ASD in the population have been carried out around the world. In studies with a sample size of over 100,000 children, the following prevalence rates were found (in relation to 10,000) in Hong Kong 16.1 [12], in the United Kingdom, 24 [13], in France, 36.5 [14], in Canada, 43.1 [15] in Australia, 102.5 [16], and in Sweden, 115 [17].

The increase in prevalence is attributed to the change in the definition of ASD (the transition from DSM4 to DSM5), an increase in community awareness of ASD, and the lowering of sociocultural stigma. In addition, many studies examine hereditary or environmental risk factors for ASD.

The high prevalence rates of ASD have implications for public health in adapting to the needs of children and adults with ASD. In addition, the treatment of ASD causes a financial burden on the patients’ families, and state institutions. The burden increases when cognitive impairment accompanies ASD.

The estimated burden for the patient is between $50,000–$100,000 in the US (mainly depending on the cognitive level of the child with ASD). It was also found that the cost of supporting a person with autism throughout his or her life is estimated to be around $2.4 million in the United States and around $2.2 million in the UK. ASD represents a significant financial burden mainly due to the need to look after the child and then the adolescent and adult who cannot become an independent adult. The burden includes medical expenses such as diagnosis and treatment, educational and employment frameworks, and a decrease in family income as a result of treating a child with ASD [18]. In a cross-sectional study conducted in Israel in 2015, it was found that the private out-of-pocket expenditure per year for the treatment of a child with ASD reaches an average of $8239 [19]

MDD was found to be more prevalent in children with ASD in the past [20,21]. A large body of studies conducted in recent years has found gross motor deficits in children with ASD, including balance, gait, movement planning, and fine motor skills [22]. In the literature, an opinion is beginning to emerge that reference should be made to motor characteristics as one of the core symptoms and as a criterion for diagnosing ASD [23]. In recent years, several studies have been conducted to test whether MDD in infancy can be an early marker for ASD [24].

Early intervention in children with ASD is important [25]; therefore, indicators for clinical detection such as MDD should be explored [26]. Despite a constant rise in the evidence supporting motor delays and motor immaturity as an integral and significant issue in children with ASD and the importance of early detection and intervention in ASD, no motor scale has been specifically developed for this population. Tools that have been used to evaluate motor development in children with ASD are the Miller Function and Participation Scales (M-FUN), the Peabody Developmental Motor Scales, Second Edition (PDMS-2) [27], and the Movement Assessment Battery for Children-2 (MABC-2) [28]. Other tools evaluating motor development at infancy are the Ages and Stages Questionnaire, 3rd edition (ASQ-3), and Bayley Scales of Infant and Toddler Development, 3rd edition (BSITD-3) [24]. However, the most used measurement tool for assessing motor development is the Alberta Infant Motor Scale (AIMS) [2]. The AIMS measurement tool was developed in Canada in 1992 based on data from 506 infants [2]. During the AIMS test [29], a total of 58 motor milestones are assessed until independent walking is achieved. These milestones are observed across four different positions:

- Supine lying—a variety of supine positions, bringing the baby’s hands to the knees or feet, and rolling from the back to the stomach.
- Lying on the stomach—starting with lifting the head from the surface at different heights, reaching out with arm support, pivoting, belly crawling, four points kneeling, and reciprocal crawling.
- Sitting—from sitting the baby with a variety of supports to sitting without the support of the arms.
- Standing—starting with support standing, pulling to standing, standing and cruising with the support of objects, and ending with independent standing and walking.

The tester marks on the test the performance that was observed, and, accordingly, a raw score is given. This score is compared to the baby’s age in months, and, accordingly, the percentage to which his or her motor development corresponds is determined. Typical motor development is described as results above the 25th. Suspicion of MDD was determined between the 25th and 6th percentiles and MDD was determined at the 5th percentile and below [4]. The test has a high level of reliability [30] and sensitivity [31], and it is used as a tool to estimate motor development among developmental physicians, physiotherapists, and occupational therapists [32]. A 2012 study using AIMS examined 48 infants equally divided between high-risk ASD (due to ASD sibling) and low-risk ASD infants. The study found that the AIMS scores of at-risk infants were significantly lower than those of non-at-risk infants; however, no outcome information was reported [33].

This paper is based on a historical cohort study conducted at the Meuhedet Health Services (MHS). MHS is Israel’s third largest health fund, serving over 1.2 million (14% of the national population) clients nationwide. Ethical approval was provided by the MHS IRB as a “retrospective survey of health records without intervention”. The study objectives were to evaluate MDD and other characteristics as indicators for ASD. The objective of the current study is to present the AIMS scale results of infants who received developmental physiotherapy (DPT) services due to a diagnosis of MDD in infancy and were later diagnosed with ASD.

## 2. Method

The historical cohort study included 240,299 children who were born between 2011 and 2017 and are members of the MHS.

Inclusion and exclusion criteria—The original data file consisted of the health records of 279,510 children, MHS members, who were born between the years 2011 and 2017.

According to the State Health Law of the State of Israel, it is possible to switch between four health funds (such as MHS) every 3 months. Children who joined the MHS after the age of 24 months (N = 39,206) were excluded from the study. Another 5 children with incorrect MDD developmental data or with no documentation in the child’s file were also excluded. 

In the first stage, distribution was made to children with or without a background of MDD in infancy (up to 2 years of age). The MDD variable was retrieved from the administrative record of a development physiotherapy visit (DPT) at the Child Development Institute of MHS. In the second stage, a second division was performed for children with or without an ASD diagnosis. A child who was diagnosed with one of the ICD (International Classification of Diseases) codes was defined as an ASD case. The odds ratio for ASD was calculated. In the third stage, an individual entry was made in the child’s files with a background of MDD with an ASD diagnosis for the purpose of characterizing the children’s primary characteristics. Data extracted from the child’s files included the number of children in the family, week of birth, type of birth (normal, cesarean, or vacuum), birth weight, Apgar scores, reason for referral to a DPT, and AIMS test score. The assessments of AIMS were performed by DPTs who had been trained in the use of it. The evaluations were carried out in accordance with the instructions for using the tool as published by the developers of the tool [29]. Data were cross-referenced between AIMS scores and the reason for referral, and, in addition, an AIMS score was cross-referenced for the age at first visit to DPT. Statistical Data analysis was performed with IBM SPSS Statistics for Version 25.0., Armonk, NY, USA, IBM Corp.

## 3. Results

**Figure 1:**
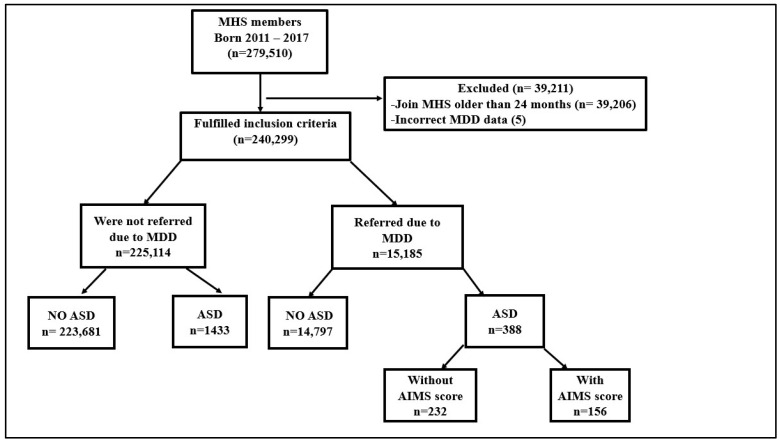
Flow chart of the research process: Criteria for exclusion, first division according to backgrounds of MDD and without background of MDD, second division according to ASD diagnosis, and final division according to recorded AIMS score. MHS—Meuhedet Health Services, MDD—motor developmental delay, ASD—Autism spectrum disorder, AIMS—Alberta Infant Motor Scale.

As shown in Figure 1, out of the original cohort of 240,299 MHS children born between 2011 and 2017, 15,185 came to DPT due to MDD. Of these, 388 children were subsequently diagnosed with ASD, and their description can be seen in Table 1. The odds ratio for ASD in children with a background of MDD was 4.1 (95% CI 3.6–4.6).

**Figure 2:**
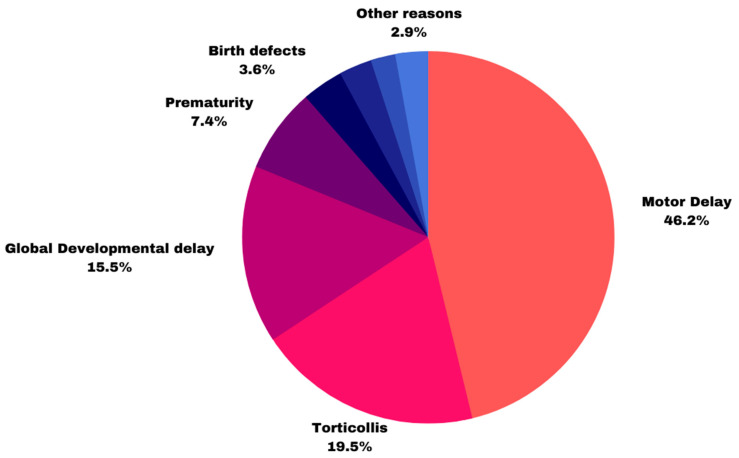
Indication for referral to DPT.

Indication for referral to DPT, shown in Figure 2, based on the initial doctor’s referral or the medical file documentation. 48 infants had 2 reasons listed for referral, while 15 had no documentation of referral in their medical files. Of the 420 referrals, 194 infants were referred due to motor delay, 82 due to torticollis, 65 due to developmental delay, 31 due to preterm birth, 15 due to birth defects, 12 due to hypertonia, and 12 due to other reasons. Nine infants were referred due to communication difficulties.

## 4. AIMS Score

**Figure 3:**
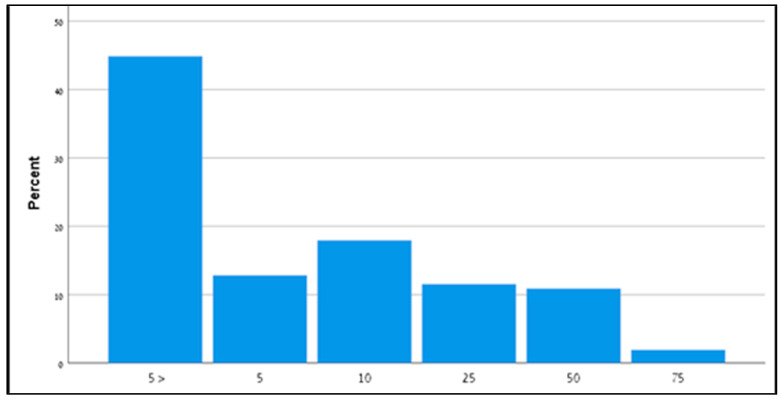
Alberta Infant Motor Scale score of 156 infants later diagnosed with ASD. AIMS—Alberta Infant Motor Scale.

Of the 388 children diagnosed with ASD, 156 (40%) had a record of AIMS test results in their medical file. As shown in Figure 3, no child in the cohort presented age-appropriate milestone acquisition. Seventy infants (45%) had an AIMS score below the fifth percentile, 20 infants (13%) had an AIMS score of 5%, 28 infants (18%) had an AIMS score of 10%, and the remaining 38 infants (24%) had an AIMS score of 25% or higher.

Cross-referencing data (shown in Table 2) between the 156 child files in which the AIMS score was recorded and the reason for the referral found that 91 (58%) of the infants were referred due to motor delay, of whom 69 (76%) had a 5th percentile or lower score, 20 (22%) infants had an AIMS score between the 6th and 25th percentiles, and 2 had a 50th percentile score. Forty-two infants (27%) were referred due to torticollis, of whom 9 (21%) had a 5th percentile or lower score, 24 infants had an AIMS score between the 6th and 25th percentiles, and 9 (21%) had a score above the 25th percentile. Fifteen infants (10%) were referred due to a general developmental delay, of whom 10 (66%) had a 5th and 4 (27%) above the 25th percentile score.

Cross-referencing data between AIMS scores and the age of the first DPT session found that 52 infants attended the first DPT session by the age of 6 months, of whom 20 (39%) had a 5th percentile or lower score and 24 (46%) had an AIMS score between the 6th and 25th percentiles. Fifty-seven infants attended the first DPT session between the ages of 7 months and 1 year, of whom 39 (68%) had a 5th percentile or lower score and 13 (22%) had an AIMS score between the 6th and 25th percentiles. Forty-seven (30%) infants attended the first DPT session between the ages of 1 and 2 years, of whom 31 (66%) had a 5th percentile or lower score, and 9 (19%) had an AIMS score between the 6th and 25th percentiles.

The rates of preterm birth (birth before week 37) and infants born with low birth weight (LBW—below 2500 g) in the MDD + ASD group were 17.1% and 18.1%, respectively.

In the description of pregnancy (N = 388), 81.7% of pregnancies were defined as normal, 17% were defined as at-risk pregnancies, and 1 pregnancy was through a surrogate mother. The number of children in the family (N = 387) had a mean of 2.57 (SD = 2.07). Fourteen of the infants were registered under a single mother (3.6%), and 25 of the infants were registered as twins (6.4%).

## 5. Discussion

This retrospective cohort study was designed to examine the association between motor delay in infancy and a later diagnosis of ASD. The association between MDD and ASD was an odds ratio of 4.1 (95% CI 3.6–4.6), which corresponds with previous studies and constitutes another supportive milestone in recognizing motor delay in infancy as an early marker for autism [21,26,34,35,36]).

Functional MRI scans showed a variety of changes in different structures in the brain of children with ASD compared to a control group that presented typical development. The changes include an increase in brain volume before the age of six, an increase in the volume of the frontal and temporal lobes, a decrease in the volume of the cerebellum and corpus callosum, and changes in the volume of the hippocampus, amygdala, and basal ganglia [37]. Focal cortical dysplasias were also found in the cerebellum [38]. The cerebellum is responsible for controlling balance, coordination, and muscle tone. Therefore, structural changes in it lead to motor difficulty [39].

The use of the AIMS test is recommended when suspecting a motor delay. Many studies have examined the use of AIMS in different countries, and it has been found that there is need to adjust the development norms according to the country. Studies conducted in Belgium [40] and the Netherlands [41] have recommended adjusting correlation values to AIMS to identify at-risk babies. This is due to lower scores than Canadian babies (where AIMS was first established). In contrast, the norms of babies from Greece [42] and Turkey [43] were found to be the same as the norms from Canada.

AIMS is used to assess motor development among different populations, including premature infants [44] and Down syndrome babies [45]. The results of the current study add children diagnosed with ASD to these populations.

From the results of the AIMS test, most of the infants (87%) had an MDD-compatible score and a suspicion of MDD. Cross-referencing the data with the reason for referring to the AIMS score found that among the infants referred due to motor delay, most of them (98%) had an MDD-compliant score and a suspicion of MDD. In contrast, these rates decreased among infants referred due to torticollis and Global Developmental Delay. Of the 42 infants referred due to torticollis, 9 (21%) had an AIMS score above the 25th percentile, defined as typical motor development. Torticollis is a condition of asymmetrical head tilt, congenital or acquired, which may appear with head rotation to the opposite side [46]. In a study that examined infants with torticollis approximately 1 year after starting treatment for DPT, it was found to be a risk factor for MDD [47]. Cross-referencing the data between the age of the first visit to DPT and AIMS scores shows that the MDD rate was relatively low among infants up to 6 months of age (39%) compared with subsequent age groups. This may be because those infants did not yet have an appropriate MDD score due to their young age and, therefore, did not accumulate significant developmental gaps.

Recent studies have found that the use of home videos is useful as observation by a professional in the AIMS assessment [48]. Also, given appropriate training, the quality of the videos was found to be high and have interrater reliability, which has the potential to use AIMS in telemedicine for babies at risk of MDD.

The key strength of this study is the use of the AIMS by DPT. DPTs are trained to detect atypical movement patterns. Usually, motor delay appears before the age of 1 year, while language delay usually appears in the second year of life. The DPT is the first professional in the child development system who treats the youngest ages [49]. Besides intervention, the place of DPT in early detection [50] of children with ASD has risen in recent years [51]. Despite this, DPTs (like other professionals, e.g., occupational therapy and speech therapy) lack specific training for early detection of ASD in infancy [52] A study conducted in Israel among pediatric health professionals found a lack of knowledge regarding early detection, regardless of professional affiliation. Although experience in treating ASD contributed to general knowledge, it did not affect knowledge in the field of early detection [53]. However, in a pilot study conducted by the same group, it was found that a DPT workshop on early ASD screening promotes participants’ level of knowledge and clinical self-efficacy [54].

Many studies have found that early and intensive therapeutic and behavioral interventions lead to good outcomes among children with ASD in the cognitive, verbal, and behavioral domains [25,55]. Early intervention underscores the importance of early diagnosis of ASD. There is a wide range of tests for toddlers who are defined as at high risk of being diagnosed with ASD. This offer includes medical examinations (such as hearing examinations), developmental evaluation of professionals, and functional, communicative, and social emphases. It is, therefore, of great importance in the early detection of infants at risk for ASD [56]. 

## 6. Conclusions

MDD can be an early marker for ASD, and, therefore, it is necessary to use standard MDD assessment tools for infants. As mentioned, AIMS is a worldwide recognized developmental assessment tool [24]. The use of AIMS as performed in this study shows that the scale can detect a high rate of MDD among infants who have subsequently been diagnosed with ASD. Its use should, therefore, be considered in the screening processes for diagnosing MDD as a possible early marker for ASD.

As a retrospective historical cohort study, the data of this research was collected from existing records or databases, which may be incomplete or inaccurate. This can limit the validity and reliability of the results. It is suggested that in the future, prospective studies should be undertaken to investigate the development of gross motor skills utilizing the AIMS in addition to early tools for evaluating language and communication, with the aim of assessing infants before they reach 1 year of age.

## Figures and Tables

**Table 1:** Description of the 388 infants with MDD and a later ASD diagnosis.

Gender	
Boys	298 (76.8%)
**Father’s age**	
Mean ± SD (years)	31.6 ± 12
**Mother’s age**	
Mean ± SD (years)	31.2 ± 6.9
**Sector**	
General Population	271 (69.8%)
Ultra-Orthodox Jews	83 (21.3%)
Israeli Arabs	34 (11%)
**Pregnancy Description**	
Risk	66 (17%)
**Age of Gestational (Weeks)**	
37>	66 (17%)
**Birth Weight (Grams)**	
2500>	70 (18.1%)
**Apgar Score (n = 226)**	
1 Min	8.4 ± 1.7
1 Min < 8	27 (6.9%)
5 Min	9.3 ± 1.1
5 Min < 9	21 (5.4%)

**Table 2:** Cross-referencing AIMS score with reason for referral and age at first DPT visit.

	Typical Motor Development >25th	Suspicion MDD 6th–25th	MDD 5th≥
General results (n = 156)	20 (13%)	46 (29%)	90 (58%)
Reason for referral			
Motor delay (n = 91)	2(2%)	20 (22%)	69 (76%)
Torticollis (n = 42)	9 (21%)	24 (57%)	9 (21%)
Global developmental delay (n = 15)	4 (27%)	1 (6%)	10 (66%)
Age of first visit DPT			
0–6 Months (n = 52)	8 (15%)	24 (46%)	20 (39%)
7–12 Months (n = 57)	5 (8%)	13 (22%)	39 (68%)
13–24 Months (n = 47)	7 (14%)	9 (19%)	31 (66%)

MDD—Motor Developmental Delay, DPT—Developmental Physiotherapy. All percentages listed in the table refer to each row on its own.

## Data Availability

The data presented in this study are available on request from the corresponding author. The data are not publicly available due to Medical Confidentiality.
